# Comparative transcriptome analysis unveils the tolerance mechanisms of *Citrus hystrix* in response to ‘*Candidatus* Liberibacter asiaticus’ infection

**DOI:** 10.1371/journal.pone.0189229

**Published:** 2017-12-12

**Authors:** Yan Hu, Xi Zhong, Xuelu Liu, Binghai Lou, Changyong Zhou, Xuefeng Wang

**Affiliations:** 1 National Citrus Engineering Research Center, Citrus Research Institute, Southwest University, Chongqing, P. R. China; 2 Ganzhou Bureau of Fruit Industry, Ganzhou, Jiangxi, P. R. China; 3 Guangxi Key Laboratory of Citrus Biology, Guangxi Academy of Specialty Crops, Guilin, Guangxi, P. R. China; South China Agricultural University, CHINA

## Abstract

Citrus Huanglongbing (HLB), a highly devastating citrus disease, is associated with ‘*Candidatus* Liberibacter asiacitus’ (*C*Las), a member of phloem-inhabiting *α-proteobacteria*. HLB can affect all cultivated citrus and no cure is currently available. Previous studies showed that Kaffir lime (*Citrus hystrix*), primarily grown in South Asia and Southeast Asia, was tolerant to HLB but the molecular mechanism remains unknown. In this study, gene expression profiling experiments were performed on HLB-tolerant *C*. *hystrix* and HLB-susceptible *C*. *sinensis* three months after inoculation with *C*Las using RNA-seq data. Differentially expressed genes (DEGs) in the two citrus cultivars were mainly involved in diverse cellular functions including carbohydrate metabolism, photosynthesis, cell wall metabolism, secondary metabolism, hormone metabolism and oxidation/reduction processes. Notably, starch synthesis and photosynthesis process were not disturbed in *C*Las-infected *C*. *hystrix*. Most of the DEGs involved in cell wall metabolism and secondary metabolism were up-regulated in *C*. *hystrix*. In addition, the activation of peroxidases, Cu/Zn-SOD and POD4, may also enhance the tolerance of *C*. *hystrix* to *C*Las. This study provides an insight into the host response of HLB-tolerant citrus cultivar to *C*Las. *C*. *hystrix* is potentially useful for HLB-tolerant/resistant citrus breeding in the future.

## Introduction

Citrus is an important economic crop in tropical and sub-tropical regions worldwide. Global citrus production in 2014 exceeded 121 million metric tons (http://www.fao.org/economic/est/est-commodities/citrus/en/), ranked the top among all the fruit crops. Citrus Huanglongbing (HLB, yellow shoot disease, also known as citrus greening disease) is one of the most destructive diseases in citrus production [1]. The disease is associated with three species of non-culturable, phloem-limited, α-Proteobacteria: ‘*Candidatus* Liberibacter asiaticus’, ‘*Ca*. L. africanus’, and ‘*Ca*. L. americanus’ [2, 3]. Among the three species, ‘*Ca*. L. asiaticus’ (*C*Las) is the most prevalent in major citrus-producing regions, e.g. China, Brazil and the United States.

No effective cure is currently available for HLB-infected citrus plants. Integrated management practices including the use of pathogen-free nursery stocks, control of insect vectors and removal of infected trees are the commonly recommended strategies. Although all commercial citrus cultivars are susceptible to HLB, a few HLB resistance or tolerance citrus and citrus relativeshave been reported [4–6]. Kaffir lime (*C*. *hystrix*), primarily grown in South and Southeast Asia, has been used for cooking and traditional medicine. Extracts of Kaffir lime leaves, rich in terpenoids and phenolic contents, could inhibit the proliferation of cancer cells and biofilm formation of bacteria [7, 8]. *C*. *hystrix* was experimentally validated as HLB-tolerance citrus cultivar in Malaysia [4, 9]. Similar result was also obtained in our laboratory based on three-year biological indexing and qPCR monitoring (unpublished data).

To better understand the HLB resistance and tolerance mechanisms in citrus, substantial research efforts have been made to study citrus-*C*Las interaction through microarray and high-throughput sequencing technology [10–16]. Gene expression profiles of different citrus leaves, fruits and roots from susceptible citrus cultivars infected with *C*Las showed that genes involved in sugar and starch metabolism, photosynthesis, cell wall metabolism, stress response and hormone signaling were significantly altered [10–16]. Comparative transcriptional and transcriptome studies between HLB tolerant and susceptible citrus cultivars were performed to identify genes of HLB tolerance to HLB (17–19). The HLB tolerance breeding line US-897 (*C*. *reticulata*×*P*. *trifoliata*) might be associated with the constitutively higher expression of defense-related genes, compared with the susceptible ‘Cleopatra’ mandarin (*C*. *reticulata*) [17]. Comparative transcriptional analysis of tolerant rough lemon (*C*. *jambhiri*) and susceptible sweet orange (*C*. *sinensis*) in response to *C*Las infection revealed more differentially expressed genes in HLB-infected rough lemon than those in sweet orange at early stages but substantially fewer at late time points. Genes coding for cell wall proteins, β-1, 3-glucanases, and GASA1, were identified as potential HLB tolerant citrus improvements program [18]. Recently, expression differences in secondary metabolites, pathogenesis related genes, transcription factors, hormone signaling pathways and receptor-like kinases were found from ‘Jackson’ grapefruit-like-hybrid and susceptible ‘Marsh’ grapefruit (*C*. *paradisi*) [19].

In light of the successful examples above, this study was to study transcriptical difference between highly susceptible sweet orange trees and tolerant kaffir lime trees using RNA-seq approach. The identification of differentially expressed genes in the two citrus cultivars at an early stage of HLB infection provides new information for future HLB management.

## Materials and methods

### Plant materials

The disease free budwoods of tolerant kaffir lime (*C*. *hystrix*) and susceptible pineapple sweet orange (*C*. *sinensis*) collected from virus-free citrus repository of National Citrus Virus Exclusion Center (NCVEC) in Southwest University were grafted on two-year-old Carrizo citrange rootstocks (*C*. *sinensis*×*P*. *trifoliata*), respectively. The budwood used for inoculation was Guanximiyou pummelo (*C*. *grandis*) solely infected with *C*Las strain GZBJT maintained in greenhouse in NCVEC. Three replicate trees per genotype were infected with *C*Las through bud grafting, with three *C*Las-free bud grafted trees as the control.

### DNA extraction and ‘*Ca*. L. asiaticus’ detection

Fully expanded mature leaves were collected from diseased and mock-inoculated trees every twenty days for DNA extraction described previously [20]. Both conventional PCR using primer set OI1/OI2c and TaqMan qPCR assays with an iCycler IQ5 (BioRad, Hercules, CA) were used to detect *C*Las [2, 21].

### RNA extraction and RNA-sequencing

Once infection had been confirmed by PCR and qPCR, total RNA were extracted from fully expanded young leaves with mortar and pestle in liquid nitrogen. RNA extraction process was performed according to TRIzol protocol (Invitrogen, Carlsbad, CA). The concentration of total RNA was determined by Nanodrop 2000 Spectrophotometer, and the RNA integrity number (RIN) was evaluated by Agilent 2100 Bioanalyzer. Three RNA replicates of each treatment were pooled in equal amount, and sent to Beijing Genomics Institute (BGI)-Wuhan (China) for RNA sequencing.

### Data analysis

After cleaning the raw reads, we mapped the clean reads to *Citrus sinensis* genome (http://www.ncbi.nlm.nih.gov/genome/ 10702) using Bowtie 2 [22] and then calculated gene expression level with RSEM [23]. Differentially expressed genes (DEGs) were screened based on PossionDis with a cutoff threshold FDR of 0.001 and log_2_ fold change (FC) of ≥ 1.00 or ≤ -1.00.

Functional enrichment analysis was carried out using the GO database (http://www.geneontology.org/). The DEGs were also analyzed by PageMan [24] embedded in MapMan [25]. A Wilcoxon test was applied and a statistics-based overview of changed pathways from global gene expression alterations was provided. The same data sets were also imported into MapMan to map the differentially expressed genes into specific pathways.

### RT-qPCR validation

To verify the results of RNA-seq, 16 DEGs involved in carbohydrate metabolism, photosynthesis, cell wall metabolism, secondary metabolism, hormone metabolism and Pathogenesis related (PR)-proteins were selected for RT-qPCR analysis. The same batch RNA samples used in RNA-seq analysis were used for RT-qPCR. The first strand cDNA was synthesized using HiScript^®^ Reverse Transcriptase (Vazyme, Nanjing, China) and 6N random primers according to the manufacture’s protocol. RT-qPCR was performed on an iCycler IQ5 instrument with SYBR^®^ Premix Ex Taq^™^ II (TakaRa, Dalian, China). The cycling conditions included incubation for 30 s at 95°C followed by 40 cycles of amplification (95°C for 5 s and 60°C for 25 s). *GAPDH* (Glyceraldehyde-3-Phosphate Dehydrogenase) was selected as the internal reference gene [26] and the relative expression values were calculated with Ct method (2^-ΔΔCt^). The primers used in RT-qPCR are listed in S1 Table.

## Results and discussion

### Identification of differentially expressed genes

**Fig 1:**
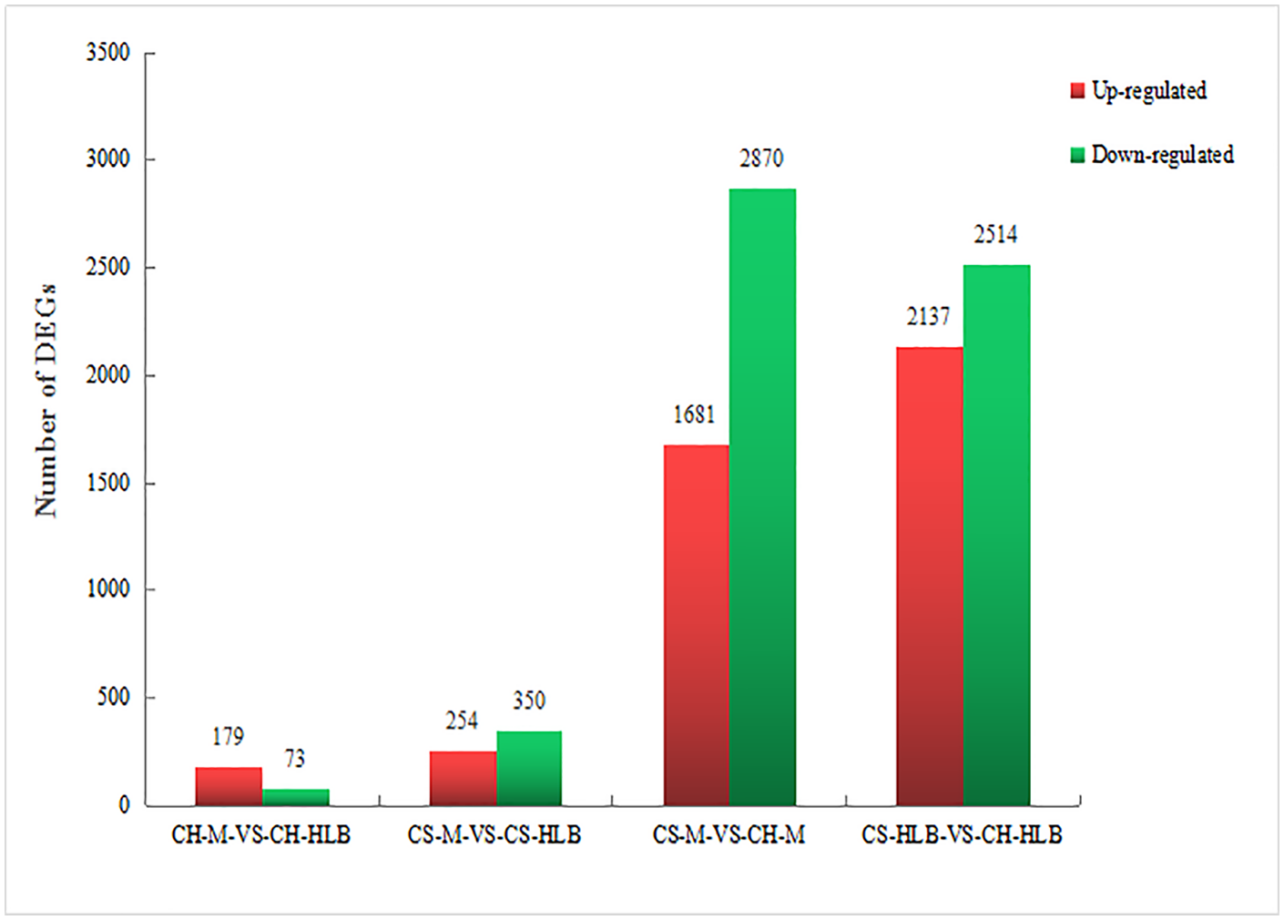
Statistic of differentially expressd genes (DEGs) of different citrus cultivars in response to *C*Las. CH, *Citrus hystrix*; CS, *C*. *sinensis*; M, Mock/healthy; HLB, Huanglongbing; CH-M-VS-CH-HLB, DEGs in HLB-infected *C*. *hystrix* compared with healthy control; CS-M-VS-CS-HLB, DEGs in HLB-infected *C*. *sinensis* compared with healthy control; CS-M-VS-CH-M, DEGs between healthy *C*. *hystrix* and healthy *C*. *sinensis*; CS-HLB-VS-CH-HLB, DEGs between HLB-infected *C*. *hystrix* and HLB-infected *C*. *sinensis*.

Around 44 million clean reads were obtained from infected and control plants and mapped to *Citrus sinensis* genome [27], with approximately 62% success for *C*. *hystrix*, and 77%-78% for *C*. *sinensis* libraries (S2 Table). Dramatic differences between the transcriptome profiles of *C*. *hystrix* and *C*. *sinensis* were observed in response to *C*Las infection. Overall, the number of differentially expressed genes (DEGs) was greater in susceptible sweet orange than in tolerant kaffir lime when compared to their respective healthy control. Among these DEGs, 179 genes were up-regulated and 73 were down-regulated in *C*. *hystrix*, while 254 genes were up-regulated and 350 were down-regulated in *C*. *sinensis* (Fig 1). Due to the differences in genetic background between the two citrus cultivars, the DEG number of infected/healthy *C*. *hystrix* in relation to infected/healthy *C*. *sinensis* was much greater than the number of the two infected cultivars in relation to their mock-inoculated healthy controls (Fig 1).

### Gene ontology classification of DEGs

**Fig 2:**
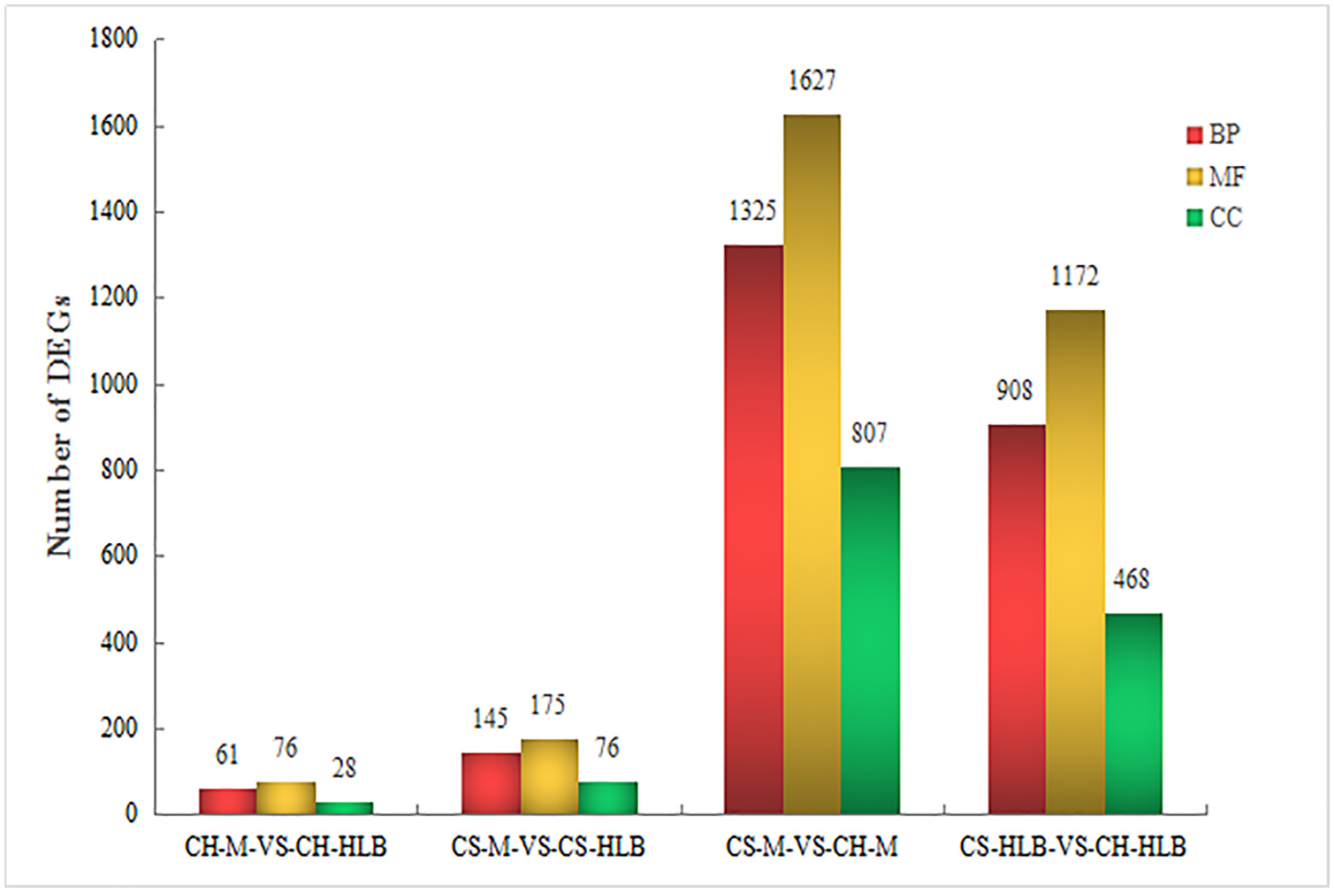
Gene ontology classification of differentially expressed genes in different citrus hosts in response to ‘*Candidatus* Liberibacter asiaticus’. BP, Biological Process; CC, Cell Component; MF, Molecular Function.

The DEGs were enriched into different function categories through Gene ontology (GO) enrichment analysis (Fig 2). Significantly enriched GO terms were extracted using TopGO [28]. For DEGs in HLB tolerant *C*. *hystrix*, the most significantly enriched GO terms in biological process were related to ‘threonine metabolic process’ (GO:0006566), ‘negative regulation of peptidase activity’ (GO:0010466), ‘regulation of proteolysis’ (GO:0045861), and ‘regulation of peptidase activity’ (GO:0052547). The most significant GO terms in molecular function included ‘terpene synthase activity’ (GO: 0010333), and ‘carbon-oxygen lyase activity, acting on phosphates’ (GO:0016838). No GO term in cellular component was enriched in DEGs in *C*. *hystrix* (S2 Table). For DEGs in susceptible *C*. *sinensis*, only two GO terms in biological process, ‘reactive oxygen species metabolic process’ (GO: 0072593) and ‘cellular protein complex assembly’ (GO: 0043623) were enriched. The most significant GO terms in molecular function were related to ‘transferase activity’ (GO: 0016758), ‘cellulose synthase activity’ (GO: 0016759), and ‘glucuronosyltransferase activity’ (GO: 0015020). Only three GO terms in cellular component were enriched in DEGs in *C*. *sinensis* and the most significant one was ‘external encapsulating structure’ (GO: 0030312) (S3 Table).

DEGs within two infected cultivars were mainly enriched in nine biological process GO terms, including ‘drug transport’ (GO: 0015893), ‘response to drug’ (GO: 0042493) and ‘response to chemical’ (GO:0042221). Besides, nine molecular function GO terms were enriched, such as ‘catalytic activity’ (GO: 0003824), ‘oxidoreductase activity’ (GO: 0016491), ‘transferase activity’ (GO: 0016758, GO: 0016757), whereas only one cellular component GO term, ‘intrinsic component of membrane’ (GO: 0031224), was enriched in DEGs in the two cultivars in response to *C*Las (S4 Table).

### Gene pathway enrichment analysis of *C*Las-modulated host pathways

**Fig 3:**
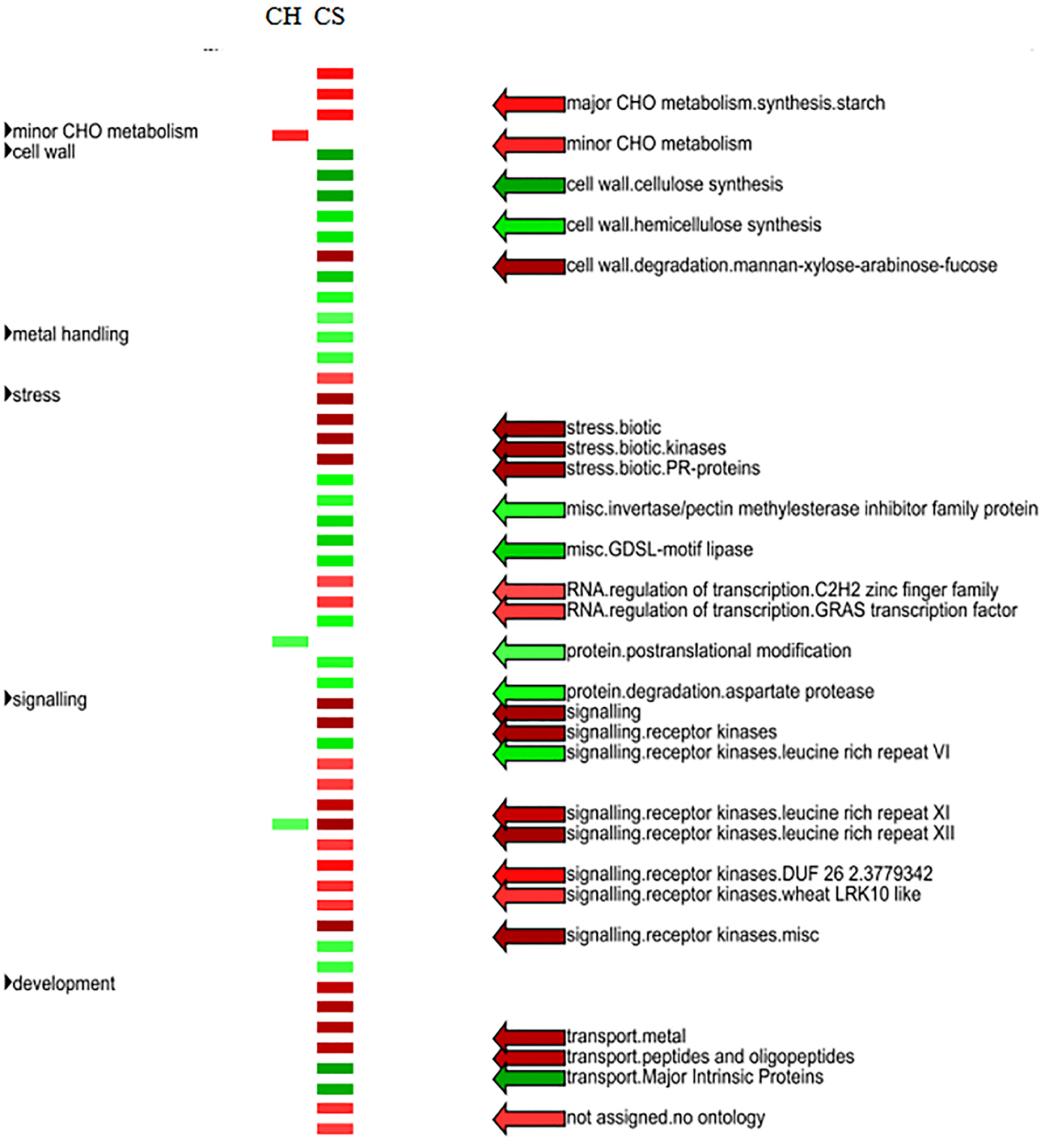
Comparative PageMan display of perturbed pathways in *C*Las-affected *C*. *hystrix* and *C*. *sinensis*. The log_2_ fold change of gene expression (mock-inoculated controls versus CLas-inoculated plants) was input into PageMan and subjected to a Wilcoxon test. Results were shown as a false-color heat-map-like display. Significantly up-regulated pathways are colored in red, while those colored in green are significantly down-regulated. Pathways without significant changes are white. Names of pathways are indicated on the right panel. CH, CH-M-VS-CH-HLB; CS, CS-M-VS-CS-HLB.

PageMan analysis showed that only three pathways significantly enriched were associated with the DEGs identified in *C*. *hystrix* (Fig 3). Pathways related to major carbohydrates (CHO) metabolism was up-regulated, while pathways related to posttranslational modification and LRR XII receptor kinases were down-regulated in tolerant citrus plants. Instead, much more pathways were significantly enriched in *C*. *sinensis* (Fig 3). The up-regulated pathways were starch synthesis, cell wall degradation, stress biotic, regulation of transcription, receptor kinases signaling, and metal transport in susceptible citrus plants. The down-regulated pathways included cellulose synthesis, hemicellulose synthesis, invertase/pectin methylesterase inhibitor family protein, GDSL motif lipase, and aspartate protease mediated protein degradation.

**Fig 4:**
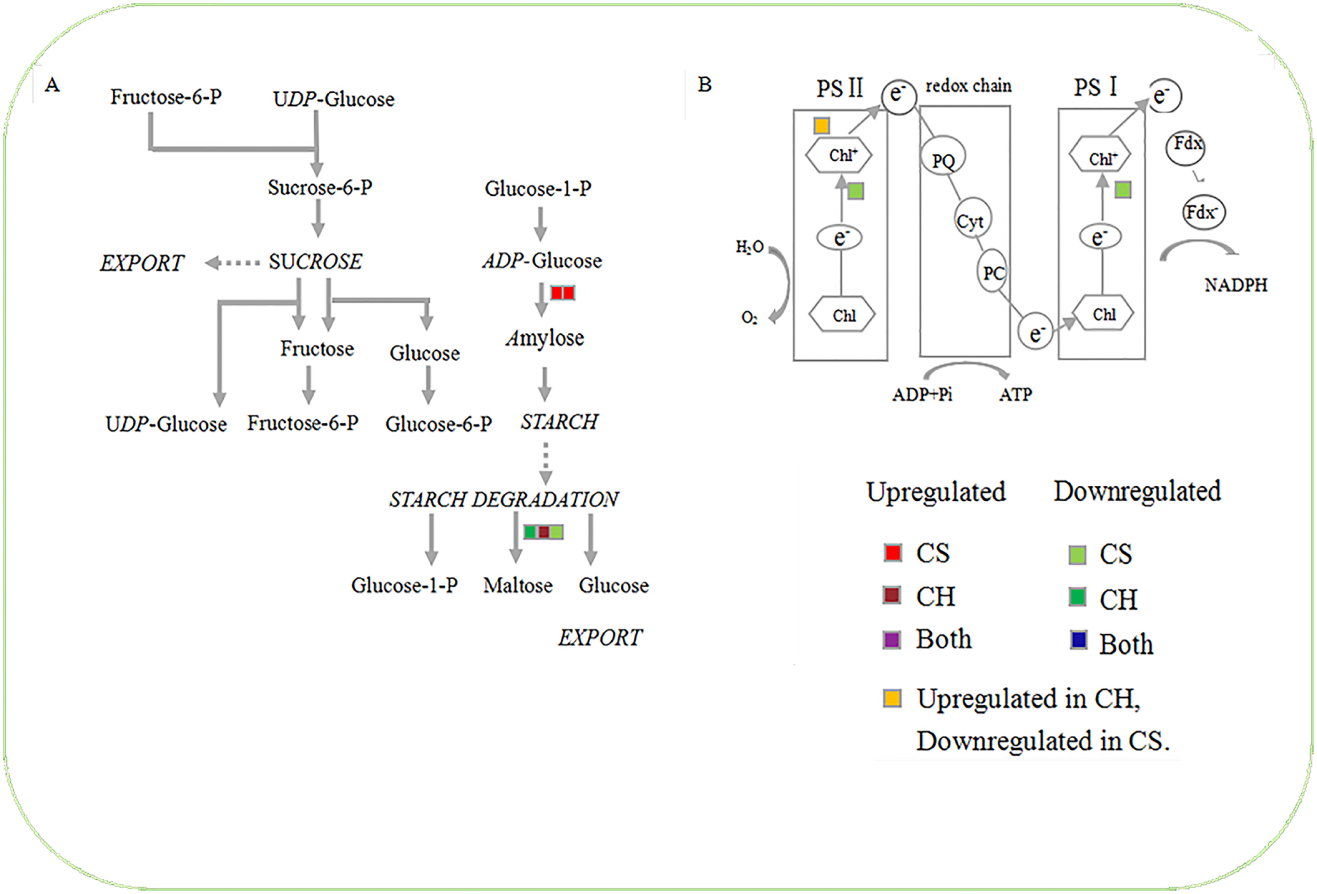
Differentially expressed genes involved in starch metabolism (A) and photosynthesis (B). Colored squares indicate up- or down-regulated genes with log_2_ fold change (FC) ≥ 1.00 or ≤ -1.00.

**Fig 5:**
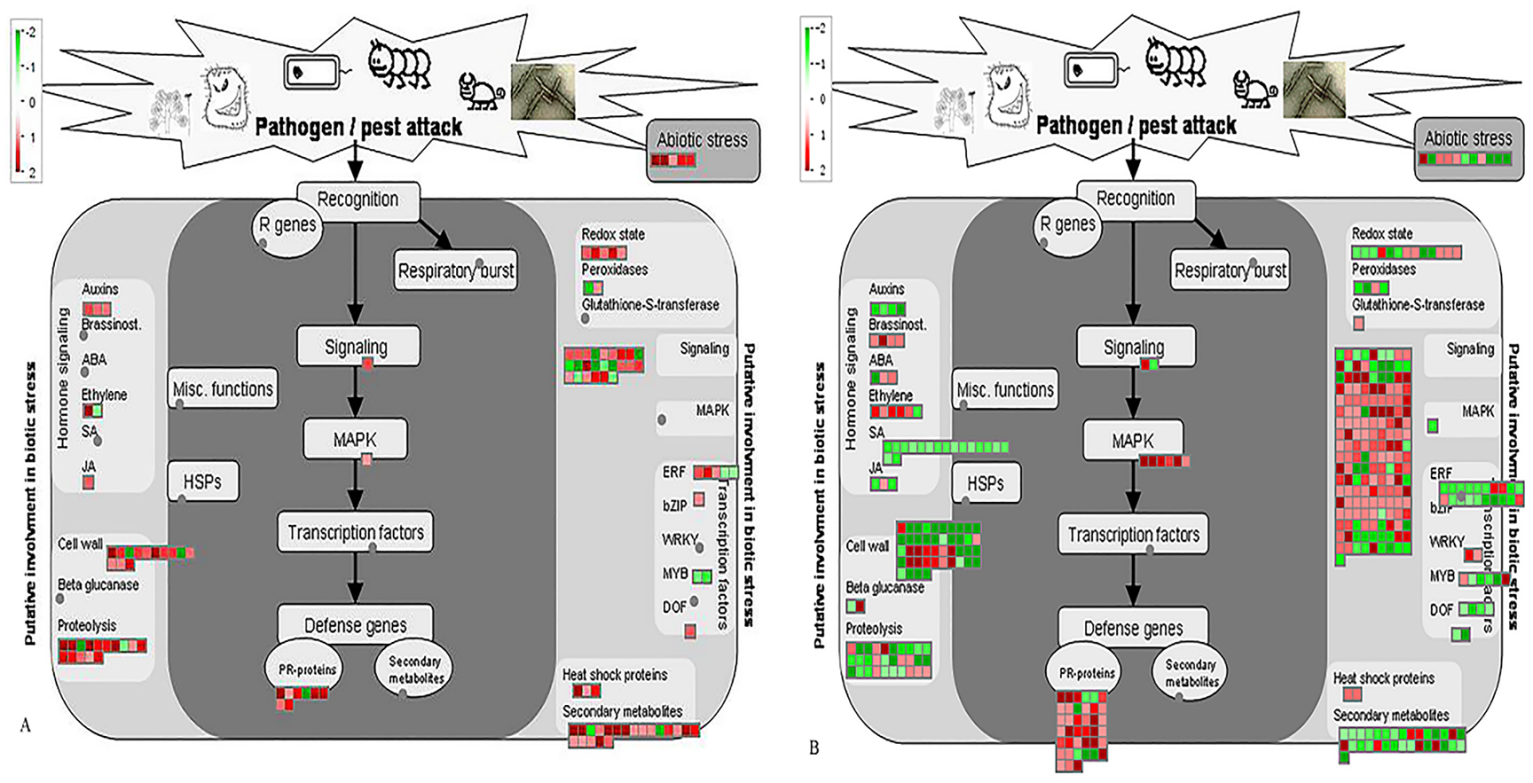
MapMan analysis of differentially expressed genes in *C*Las-affected *Citrus hystrix* (A) and *C*Las-affected *C*. *sinensis* (B) involved in stress responses. Red squares represent genes those were significantly up-regulated; green squares represent genes those were significantly down-regulated.

MapMan software was applied to display and analyze the functional classes those were significantly different in *C*Las-affected *C*. *hystrix* and *C*. *sinensis*. The results showed that DEGs were mainly involved in diverse cellular functions including carbohydrate metabolism, photosynthesis, cell wall metabolism, secondary metabolism, hormone metabolism, PR proteins and oxidation/reduction processes (Figs 4 and 5, S5 Table).

#### Carbohydrate metabolism and photosynthesis process

Cytopathology in different stage of HLB infection revealed swelling of the middle lamella between cell walls surrounding sieve elements in non-symptomatic citrus new flushes at the early infection stage and then necrosis of sieve elements and companion cells and phloem plugging by the callose-like material and excessive starch at the later infection stage [29–31]. It suggests that the disturbance of starch metabolism and the inhibition of the transport of photosynthate may contribute to HLB development in citrus hosts. Microarray or RNA-seq analysis also proved that carbohydrate metabolic process was significantly changed and photosynthesis was repressed in HLB-affected citrus [15, 17, 18, 30, 32]. From these studies, the key starch synthesis genes were generally up-regulated and the starch degrading genes were down-regulated in affected susceptible citrus. Recently, proteomic analysis suggested no clear correlation was observed from starch pathway regulation between moderately tolerant cultivar ‘Volkameriana’ and susceptible navel orange [33].

In this study, several genes involved in carbohydrate metabolism were regulated in the early stage of infection with *C*Las (Fig 4, S5 Table). The starch synthesis was induced while the starch degradation was depressed in *C*. *sinensis*. Two glycogen synthase (starch synthase, *glgA*) geneswere significantly up-regulated, whereas beta-amylase (*BAM*) gene was down-regulated in *C*. *sinensis* (Fig 4, S5 Table). However, the expression levels of these genes were not significantly changed in *C*Las-affected *C*. *hystrix*. Instead, one gene encoding alpha-amylase (*AAM*), associated with starch degradation, was slightly up-regulated in *C*. *hystrix* (Fig 4, S5 Table).

Genes encoding light-harvesting complex II chlorophyll a/b binding protein 1, Photosystem II psbP domain-containing protein 1 and photosystem I subunit O, which involved in light action of photosynthesis, were all down-regulated in *C*. *sinensis* (Fig 4, S5 Table). The three genes were not significantly changed in *C*. *hystrix*, suggesting the photosynthesis process may not be depressed in tolerant *C*. *hystrix*.

Taken together these findings showed that the repression of key proteins involved in photosynthetic light reactions and up-regulation of starch-related pathways in *C*. *sinensis* was in agreement with previous studies. Notably, no significant change in starch synthesis and photosynthesis process may play important roles in tolerance of *C*. *hystrix*. Anatomical evidences from different infection stage of kaffir limeare required to prove the transcriptome data.

#### Genes involved in cell wall and cell organization

The plant cell wall is comparable to an exoskeleton surrounding the plant cell and providing both structural support and protection from biotic as well as abiotic stresses [34]. It is well established that the plant susceptibility to pathogens depends on the cell wall composition and structure, which determines its recalcitrance to degradation by cell wall modifying enzymes (CWMEs) produced by pathogens [35]. Plant cell walls are composed of layers of cellulose microfibers embedded in a matrix of pectin and hemicellulose, plus some structural proteins [36–38]. There were 44 and 13 genes found related to cell wall metabolism among the DEGs in HLB-susceptible and tolerant cultivars, respectively (Fig 5, S5 Table). Overall, most genes involved in cellulose synthesis, cell wall proteins and cell wall modification were depressed in diseased *C*. *sinensis*. For instance, several genes encoding expansin, which are related to cell wall breakdown, including EXLA1, EXPA5 and EXLB1, were down-regulated in *C*. *sinensis*, and EXLA1 was also down-regulated in *C*. *hystrix*. Expansin related genes were reported to exhibit high expressions in susceptible ‘Marsh’ grapefruit [19]. Down-regulation of these genes in both *C*. *hystrix* and *C*. *sinensis* indicates a host defense response to *C*Las infection. A group of genes encoding xyloglucan endotransglucosylase/hydrolase proteins (XTH22, XTH23), associated with cell wall degradation, were slightly up-expressed in *C*Las-affected *C*. *sinensis*, whereas no significant difference was observed in *C*Las-affected *C*. *hystrix*. Furthermore, DEGs involved in cellulose synthesis, such as cellulose synthase A (CESA), cellulose synthase-like protein A2 (CSLA2), cellulose synthase-like protein A9 (CSLA9) and cellulose synthase-like protein C5 (CSLC5) were all down-regulated in *C*Las-affected *C*. *sinensis*, while CSLA2 and CSLA9 were up-regulated in *C*Las-affected *C*. *hystrix*. Thus, kaffir lime responded to *C*Las by activating enzymes that would work together to strengthen the cell wall, which may contribute to the reinforcement of physical barriers to restrict the invasion of *C*Las.

#### Secondary metabolism

Secondary metabolism plays important roles in plant defenses. Previous studies revealed that most genes involved in secondary metabolites, including terpenoid, flavonoid and phenylpropanoid biosynthesis pathways, were highly induced in *C*Las-affected leaves [15, 17, 19, 39], whereas most of these genes were down-regulated in roots following HLB infection [16]. Here, most DEGs involved in secondary metabolism pathway were activated in *C*. *hystrix*, but were suppressed in *C*. *sinensis*. These DEGs were categorized into the biological processes of terpene biosynthesis, flavonoids biosynthesis and phenylpropanoids biosynthesis (Fig 5, S5 Table). Terpenoids are a structurally diverse group of natural products, which function as plant antioxidants, insect attractants or repellents [40, 41]. Flavonoids are anti-fungi substances and antioxidants [42]. Phenylpropanoids serve as structural polymers including lignins, provide protection from pests and UV light and attract pollinators as pigments [43]. Two genes encoding germacrene D synthase-like (GDSL) were up-regulated in both two cultivars in response to *C*Las infection. Two genes encoding gamma-terpinene synthase (TPS2) were only up-regulated in HLB tolerant citrus trees. The expression level of one gene encoding (R)-limonene synthase 1 was increased in *C*. *hystrix*, while the other two genes related to this enzyme were suppressed in *C*. *sinensis*. GDSL, TPS2 and (R)-limonene synthase were involved in terpene biosynthesis. One gene encoding 2-oxoglutarate and Fe (II)-dependent (2OG-Fe (II)) oxygenases, which is associated with flavonoids biosynthesis, was strongly up-regulated in *C*. *hystrix*, whereas it was down-regulated in *C*. *sinensis*. Genes involved in phenylpropanoids biosynthesis such as isoflavone reductase (IRL) and cinnamoyl-CoA reductase 1 (CCR), were induced in *C*. *hystrix*, while phenylalanine ammonia-lyase (PAL) and 4-coumarate—CoA ligase 1 (4CL1), which also play an important role in phenylpropanoids biosynthesis, were suppressed in *C*. *sinensis*.

The tolerant cultivars contain relatively higher specific volatile compounds such as monoterpenes and aldehydes than susceptible cultivars [44]. It should be noted that Kaffir lime leaves contain various classes of phytochemical substances including terpenoids and polyphenolic which are known for their antimicrobial activities [7, 8]. The up-regulation of genes involved in secondary metabolism pathways, and high phytochemical substances containing in kaffir lime leaves may contribute to its tolerance to *C*Las.

#### Hormone metabolism

Phytohormones play critical roles in helping the plants to adapt to adverse environmental conditions. The elaborate hormone signaling networks and their ability of crosstalk make them ideal candidates for mediating defense responses [45]. There were 35 hormone signaling genes in identified DEGs (Fig 5, S5 Table). Auxin was found to promote the expression of expansins in plants [46–48], which contribute to breakage of plant cell walls. Of the seven auxin-related genes, three genes related to crocetin glucosyltransferase were up-regulated in *C*. *hystrix*, two auxin-responsive proteins, SAUR72 and IAA1, were down-regulated in *C*.*sinensis*. SAUR was proved to act as a negative regulator of auxin synthesis in rice and may promote resistance to pathogens [49]. Transcription analysis showed that three SAUR-like genes were up-expressed in HLB tolerant ‘Jackson’ grapefruit, which may also contribute to the down expression of expansin [19]. A gene encoding the senescence-related gene (SRG1), involved in ethylene metabolism, was highly induced in response to *C*Las in *C*. *hystrix*. The expression of ethylene-responsive transcription factor ERF07 was up-regulated in both cultivars, while ERF107 was down-regulated. Ethylene-responsive transcription factor and EIN3-binding F-box protein 1 were only up-regulated in *C*. *sinensis*. Notably, five genes involved in salicylic acid metabolism were suppressed in *C*. *sinensis* but not in *C*. *hystrix*. JA levels in response to pathogen infection clearly highlight its involvement in plant defense responses. The key enzyme involved in jasmonate synthesis, lipoxygenase 2 (LOX2) [50], were up-regulated in both cultivars. JA-responsive gene expression for defense response is mainly mediated by a transcription factor JASMONATE INSENSITIVE 1/MYC2 (JIN1/MYC2) [51]. Up-regulation of transcription factor MYC2 was only observed in *C*. *hystrix*. These results showed that JA signal transduction may be activated by infection with *C*Las, especially in *C*. *hystrix*.

#### Pathogenesis-related (PR) genes

The pathogenesis-related (PR) proteins of plants are a group of host-encoded, inducible proteins whose synthesis is often associated with certain forms of resistance to pathogens and stresses [52]. Previous studies showed that most genes related to PR-proteins were induced by *C*Las infection, including receptor-like protein kinase, TIR-NBS-LRR (Toll interleukin-1 receptor nucleotide-binding site leucine-rich repeat) class and NB-ARC (nucleotide binding-adaptor shared by APAF-1, certain R gene products and CED4) domain proteins, and Kunitz family trypsin and protease inhibitor protein [11, 18, 32, 39].

In this work, more genes related to PR genes were induced in *C*. *sinensis* than in *C*. *hystrix* (Fig 5, S5 Table). Several genes related to kinases involved in biotic stress, including LRR receptor-like serine/threonine-protein kinase GSO1, LRR receptor-like serine/threonine-protein kinase ERL2, LRR receptor-like protein kinase At1g35710, and receptor-like protein 12, were slightly up-expressed in *C*. *sinensis*. A gene encoding leucine-rich repeat receptor-like serine/threonine-protein kinase (At2g24130) was up-regulated more than 8-fold in *C*. *hystrix*, but showed no significant difference in *C*. *sinensis*. Notably, two genes encoding miraculin, a Kunitz family trypsin and protease inhibitor protein which are associated with plant defenses, were only up-regulated in *C*. *hystrix* (S5 Table). In addition, some genes related to disease resistance proteins, such as RPS4 and RPM1, exhibited higher expression level in *C*. *hystrix*. RPS4 and RPM1 act as R genes to participate in the effector-triggered immunity (ETI) process, which play an important role in plant defense response [53, 54].

#### Oxidation/Reduction processes

14 genes involved in oxidation/reduction processes were differentially regulated between the HLB-susceptible and tolerant cultivars. It should be noted that most of them were up-regulated in tolerant *C*. *hystrix* and down-regulated in *C*. *sinensis*. Up-regulation of several glutaredoxin genes such as GRXC6, GRXC9 and GRXS9, were only identified in *C*. *hystrix*. These proteins have activity of glutathione-disulfide bond oxidordeuctase, which can reduce the amount of micromolecular disulfide and proteins. A gene encoding thioredoxin M3, a thiol-disulfide bond oxidordeuctase which required for maintaining permeability of plant meristem and regulation of callose deposition in *Arabidopsis*, was inhibited in *C*. *sinensis*. Besides, a group of superoxide dismutase (SOD) and peroxidase (POD) genes were differentially expressed in both cultivars. Of them, Cu/Zn-SOD and POD4 were up-regulated in *C*. *hystrix*, whereas they were down-regulated in *C*. *sinensis* (S5 Table). Proteins involved in oxidation/reduction processes, including peroxidases and glutathione-S-transferases, are usually associated with the prevention of oxidative stress, which are induced by reactive oxygen species (ROS). Proteomic studies revealed that a higher activation of glutathione-S-transferases, which include several isozymes that help detoxify xenobiotic compounds, was observed in tolerant cultivar ‘Volkameriana’ [33]. Therefore, activation of peroxidases, Cu/Zn-SOD and POD4, may also enhance the tolerance of *C*. *hystrix* to *C*Las.

### RT-qPCR validation

**Fig 6:**
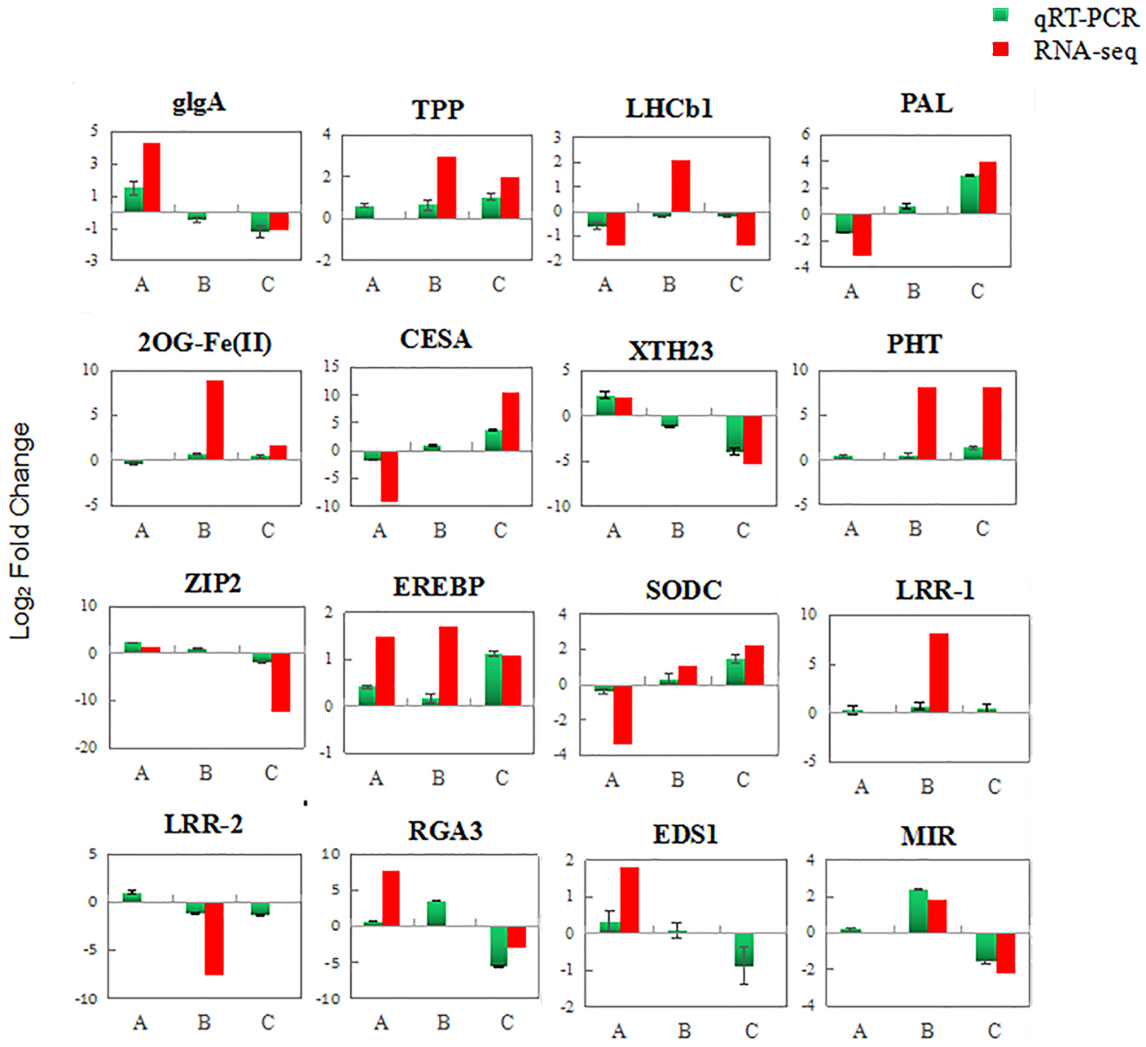
RT-qPCR and RNA-seq profiles of 16 selected differentially expressed genes (DEGs). A. DEGs between healthy and HLB-infected *Citrus sinensis*; B, DEGs between healthy and HLB-infected *C*. *hystrix*; C, DEGs between HLB-infected *C*. *hystrix* and HLB-infected *C*. *sinensis*. glgA, starch synthase; TPP, trehalose 6-phosphate phosphatase; LHCb1, light-harvesting complex II chlorophyll a/b binding protein 1; PAL, phenylalanine ammonia-lyase-like; 2OG-Fe(II), 2-oxoglutarate (2OG) and Fe(II)-dependent oxygenase; CESA, cellulose synthase A; XTH23, xyloglucan endotransglucosylase/hydrolase protein 23; PHT, phosphate transporter; ZIP2, zinc transporter 2; EREBP, ethylene-responsive transcription factor ERF017; SODC, superoxide dismutase [Cu-Zn]; LRR-1, LRR receptor-like serine/threonine-protein kinase; LRR-2, LRR-like serine/threonine-protein kinase BAM2; RGA3, disease resistance protein RGA3, NB-ARC class; EDS1 (enhanced disease susceptibility 1 protein); MIR, miraculin.

To validate the accuracy of the RNA-seq data, 16 genes classified in different function groups were tested by RT-qPCR. The expression profiles of 15 genes were consistent with the RNA-seq data, demonstrating the reliability of RNA-seq analysis. The RT-qPCR result of LHCb, encoding light-harvesting complex II chlorophyll a/b binding protein 1, did not agree with those obtained from the RNA-seq in *C*. *hystrix*. In addition, several genes showed no difference in RNA-seq data, but differentially expressed by RT-qPCR analysis. For example, glgA and XTH23 showed lower expression levels, PAL and RGA3 showed higher expression levels in *C*Las-infected *C*. *hystrix* based on qRT-PCR analysis (Fig 6).

## Conclusions

Global transcriptome profiles between the HLB tolerant *C*. *hystrix* and susceptible *C*. *sinensis* showed that specific responses in carbohydrate metabolism, photosynthesis process, cell wall metabolism, secondary metabolism and oxidation/reduction processes may play important roles against *C*Las attack in *C*. *hystrix*. 1) starch synthesis and photosynthesis process were not disturbed in *C*Las infected *C*. *hystrix*. 2) several cellulose synthase and cellulose synthase-like family proteins, which may strengthen the cell wall synthesis, were induced in *C*. *hystrix*. 3) most DEGs involved in secondary metabolism pathways were activated in *C*. *hystrix*, but were depressed in *C*. *sinensis*. 4) the activation of peroxidases, Cu/Zn-SOD and POD4, may also enhance the tolerance of *C*. *hystrix* to *C*Las, acting as detoxification proteins.

## Supporting information

S1 Table The primers used for RT-qPCR validation. (XLS)

S2 Table Gene ontology enrichment results of differentially expressed genes in *Citrus hystrix*. (XLS)

S3 Table Gene ontology enrichment results of differentially expressed genes in *Citrus sinensis*. (XLS)

S4 Table Gene ontology enrichment results of differentially expressed genes between *Citrus hystrix* and *C*. *sinensis*. (XLS)

S5 Table Differentially expressed genes in *Citrus hystrix* and *C*. *sinensis* in response to infection by *C*Las. (XLS)
